# Distribution status and habitat characteristics of the endangered freshwater fish, *Microphysogobio rapidus* (Cyprinidae)

**DOI:** 10.1080/19768354.2017.1347104

**Published:** 2017-07-10

**Authors:** Yang-Ki Hong, Ha-Cheol Sung, Myeong-Hun Ko, Keun-Sik Kim, In-Chul Bang

**Affiliations:** a Department of Life Science and Biotechnology, Soonchunhyang University, Asan, Republic of Korea; b Inland Fisheries Research Institute, National Institute of Fisheries Science, Gapyeong, Republic of Korea; c Department of Biology, Chonnam National University, Gwangju, Republic of Korea; d Division of EcoScience and Department of Life Science, Ewha Womans University, Seoul, Republic of Korea; e East Sea Research Institute, Korea Institute of Ocean Science & Technology, Uljin, Republic of Korea

**Keywords:** *Microphysogobio rapidus*, endangered endemic species, distribution, habitat characteristics, conservation

## Abstract

To investigate distribution, habitat characteristics, and current conservation status of the endangered endemic species, rapid small gudgeon *Microphysogobio rapidus* (Cyprinidae), we surveyed a total of 79 sites from the historic records (20 sites) plus additional sites (59 sites) with good habitat conditions, analyzed their sites, and compared them with historic recorded sites to reveal the factors of extinction threats and causes. We found only eight out of 79 sites in the Nam River areas. The habitats were greatly reduced and restricted compared with the historic sites, which mainly cause from habitat modification, such as various types of river renovations at the main stream and tributary streams of the Nakdong River. The present habitats are higher water temperature and more number of fish species than the absent ones, but conductivity, total nitrogen, and number of weir are lower. In addition, the present sites are lower low velocity at pool and higher mean substrate at pool. From this study, we suggest that maintaining good water quality and preventing anthropogenic impacts greatly aid conservation of the *M*. *rapidus* in South Korea.

## Introduction

Global biological diversity is rapidly diminishing as a result of human activity (Sala et al. 2000; Sutherland et al. 2009). Many scientists are calling the current crisis a mass extinction, which could result in the loss of as many as 30–50% of all species within 50 years (Pimm & Raven 2000; Thomas et al. 2004; Chivian & Bernstein 2008). In the wake of rapid industrialization, South Korea’s endemic freshwater fish populations have been greatly reduced due to human activities, including water pollution, habitat destruction, construction of large-scale dams and estuary banks, and introduction of exotic species because as freshwater ecosystem is a more or less closed system, it is closely associated with human activities (Colautti et al. 2003; Sato et al. 2010). Therefore, systematic conservation and restoration strategies with a focus on endangered species are essential to prevent the loss of South Korea’s endemic freshwater fish species (MEK 2011). In South Korea, in accordance with the Wildlife Protection and Management Act, the Ministry of Environment has designated and protected nine species of Endangered Freshwater Fish Category I and 16 species of Category II (MEK 2012). Since the early 2000s, nationally funded research has been conducted on the conservation, restoration, and breeding of endangered freshwater fish. The Red Data Book of Endangered Fishes in Korea (NIBR 2011) was released in 2011 based on the Red List categories and guidelines (size reduction, appearance range, footprint, and change tendency of populations) of the International Union for Conservation of Nature and Natural Resources (IUCN 2001), whose goal is objective selection and protection of endangered species. Thus, it is critical to understand the habitat features that may influence the distributions and population sizes of endangered freshwater fishes.

*Microphysogobio rapidus* (Cyprinidae) was designated as an Endangered Wildlife Category I endemic species by the Ministry of Environment in 2012 (MEK 2012). The species inhabited in more than 10 areas of the Nakdong River basin in the past, but now appears only in one place (NIBR 2011). Therefore, it was classified as a critically endangered species by the Red Data Book of Endangered Fishes in Korea, indicating that *M*. *rapidus* faces extreme risk of extinction in the wild (NIBR 2011). Apart from the National Ecosystem Survey by the Ministry of Environment (MEK 1997–2008), no detailed investigation on the distribution of this species has been conducted. Determining the distribution, population size, and critical habitat characteristics are the first steps necessary for the long-term conservation and management of endangered species (Ko et al. 2013).

To elucidate the factors influencing the threat of extinction, we used two types of data: presence-only and presence/absence, where presence data are eight sites that we found from this surveys and absence data are 19 sites that *M*. *rapidus* were previously present but now absent. While presence-only data show the lack of absences to counterbalance presences, presence/absence data provide an assessment of how to compare presence/absence measures (Hirzel et al. 2002). Thus, we obtained the previous presence records of *M*. *rapidus* and identified the current population distribution using field work to evaluate the status of the species. The sites where *M*. *rapidus* was not detected reflect habitat changes caused by potential threat factors and we suggest the development of protection and management plans for this species.

## Materials and methods

### Collection permit

Because *M*. *rapidus* is designated and protected as Endangered Wildlife Category I by the Ministry of Environment, we needed to obtain collection permits from the Nakdong River System Environmental Office and the Daegu Regional Environmental Office.

### Distribution status

**Figure 1.**
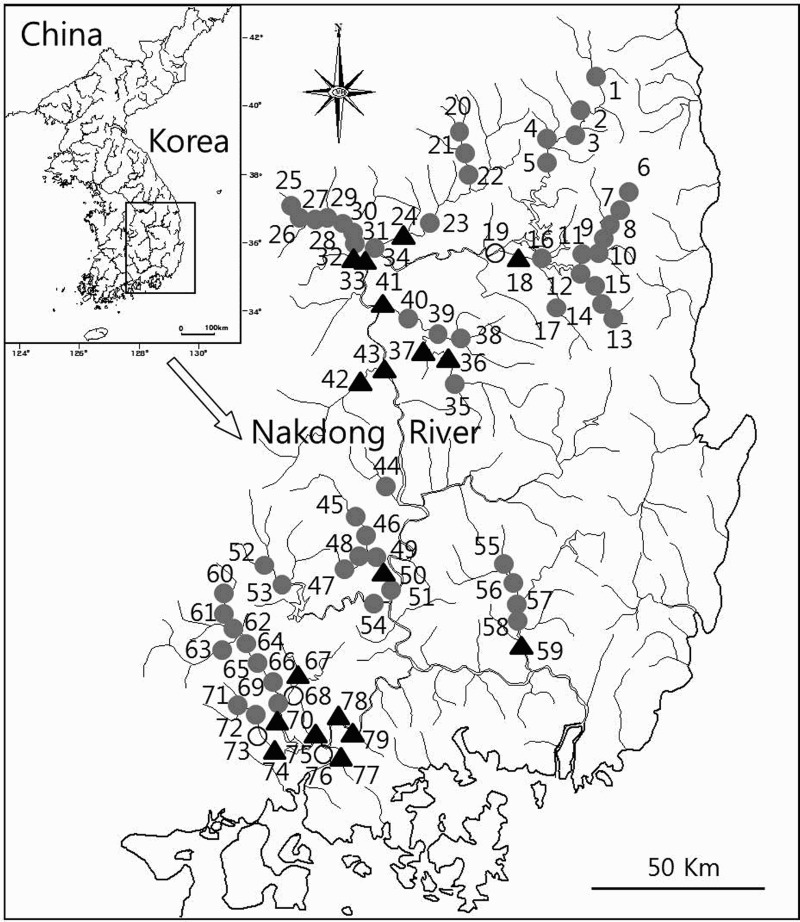
Distribution survey sites of the *M*. *rapidus* in south-eastern Korea from 1996 to 2014. Open circle: only literatures survey sites, Black triangle: literatures and present survey sites, Gray circle: only present survey sites. Data of sites is shown in Supplementary Table S1.

To investigate the previous habitat status of *M*. *rapidus*, we obtained the collection records of Chae et al. (1996, 1998a, 1998b), Kum and Yang (2002), Lee and Kim (2002), and Kang et al. (2004) along with the *M*. *rapidus*-related records in the National Ecosystem Survey by the Ministry of Environment (MEK 1997–2008), and noted the resulting distribution. The sites that previous literature (Chae et al. 1996, 1998a, 1998b; Kum & Yang 2002; Lee & Kim 2002; Kang et al. 2004) had surveyed were selected for the field study. Additional sites with good habitat conditions were also included. Thus, a total of 79 sites along the Nakdong River (including the main stream and tributaries) were studied from May 2012 to April 2014 (Figure 1 and Supplementary Table S1 online). Because *M*. *rapidus* reportedly prefers gravel substrates in the mid-lower reaches of shallow, fast-flowing streams (Chae & Yang 1999; NIBR 2011), we carried out the investigation in and around rapids, except in the winter season from November to March. We collected fish using a cast net (16 mm mesh size; 4.5 m^2^ area) 20 times and a scoop net (8 mm mesh size; 1.35 m^2^ area) for 30 min in a 100 m transect at each site. After identification, fish were released at their original capture site. The collected fish were identified according to Kim et al. (2005) and Kim and Park (2007), based on the classification system of Nelson (2006).

### Habitat analysis to identify extinction threats and causes

To analyze extinction threats and causes, we selected on presence-only eight sites and three main sites where *M*. *rapidus* was most prevalent. The flow velocity, water depth, and bottom substrate, which are fundamental elements of the microhabitat preference of fishes, were compared among sites: two sites (St. 64, 70) along the Nam River in Sancheong-gun and one site (St. 74) along the Deokcheon River in Jinju-si, where *M*. *rapidus* was most prevalent, according to the distribution survey; one site along the Yeong River in Sangju-si (St. 33) and one site along the Gam Stream in Gumi-si (St. 42), where *M*. *rapidus* used to be most prevalent but is no longer observed; and three sites, including the Yeongcheon River in Jinju-si (St. 77), which were previously preferred *M*. *rapidus* habitats but have recently been developed (Hued & Bistoni 2006; Chae & Yoon 2010). The flow velocity, water depth, and bottom substrate were measured at each site in April 2014 by dividing the rapid and pool areas that *M*. *rapidus* generally inhabits into 10 equal parts on the basis of water width. The low velocity (LV) was measured using a tachometer (Swoffer Model 2100, Seattle, WA, USA) about 5 cm above the stream bed. The water depth (WD) and bottom substrate were measured using a tape measure. The mean bottom substrate size (MS) was obtained by calculating the average of the major axes of 10 randomly selected sand, gravel, pebble, and cobble pieces from every 1 m^2^ at each site. The classification of the bottom substrate followed Cummins (1962).

The hydrological, physicochemical, biological, and other confounding factors influencing fish habitat were compared between two sets of eight sites selected from the whole 79 sites in the survey. The first set of sites included the Im Stream (St. 63), Yang Stream (St. 67), Nam River (St. 65, 66, 69) in Sancheong-gun, and three sites where many *M*. *rapidus* appeared (St. 64, 70, 74), while the other set included Banbyeon Stream in Andong-si (St. 18), Naeseong Stream in Yeocheon-gun (St. 24), Wi Stream in Uiseong-gun (St. 41), Hoe Stream in Goryeong-gun (St. 50), Miryang River in Miryang-si (St. 59), and three sites where *M*. *rapidus* did not appear during field work (St. 33, 42, 77). The river width (RW) and water width (WW), which are the hydrological environmental factors of habitats, were measured using a digital range finder (Bushnell Sport 600, Overland Park, KS, USA), and the altitude (AL) was found using Google Earth data. The physicochemical environmental factors, dissolved oxygen (DO), conductivity (CON), pH, water temperature (WT), suspended solids (SS), total nitrogen (TN), and total phosphorus (TP) were obtained from water quality monitoring network data (2004–2013) from the Water Environmental Information System of the National Institute of Environmental Research. The number of fish species (NFS) was measured as a biological factor, while the number of weirs (NW) was counted as another factor.

### Data analysis

The software package SPSS (ver. 21; IBM Corporation, Armonk, NY, USA) was used to analyze extinction threats and causes. We conducted two separate analyses because we measured different environmental variables at different places within sites. First, a one-way ANOVA was performed to compare the physicochemical, hydrological, and biological data (12 variables) between the eight presence and eight absence sites where *M*. *rapidus* was previously present but is now absent. Then, second analysis used the same methods to determine whether there was any difference between three presence and three absence sites in terms of hydrological data (6 variables) collected from 10 equal parts of each rapid and pool at every site (total 30 parts each). Before conducting the analysis, a normal distribution of environmental factor variables (Kolmogorov–Smirnov, *P* < .05) was measured. The variables without a normal distribution were included in the analysis after confirming normality through data transformation.

After that, two separate principal component analysis (PCA) were carried out to estimate the environmental factors that most influenced the habitat conditions of *M*. *rapidus*. The PCA was performed to reduce the data to independent PCs that summarized most of the variance of the original variables (Sneath & Sokal 1973). The first PCA was carried with a total of 12 variables, excluding 2 (WT and TN) with strong collinearity (Pearson’s *r* > 0.70). Then the second PCA was carried with 6 variables. The mean scores of the data for the variables were used, and the Equamax method was used to rotate the PC factor loadings. Then the PC scores for each component were compared between the presence and absence sites.

## Results

### Previous distribution (Figure 2(A))

Base on the literature, the largest numbers of *M*. *rapidus* presence sites (three sites) are in the Wi Stream and Nam River, followed by the main stream of the Nakdong, Yeong, Deokcheon River, and Yang Stream (two sites). Meanwhile, the Banbyeon, Naeseong, Gam, Hoe, Nabul, Daegok, Yeongcheon, and Miryang rivers each had one site (Chae et al. 1996, 1998a, 1998b; Kum & Yang 2002; Lee & Kim 2002; Kang et al. 2004). More than 20 individuals were collected from eight sites: St. 32 (31 individuals), St. 33 (39 individuals), St. 37 (22 individuals), St. 41 (25 individuals), St. 42 (78 individuals), St. 70 (25 individuals), St. 76 (33 individuals), and St. 79 (31 individuals). The largest number of *M*. *rapidus* appeared at St. 42. The National Ecosystem Survey by MEK (1997–2008) reported only the habitats in the Yang Stream, including St. 67 (10 individuals) and St. 68 (5 individuals).

### Current distribution (Figure 2(B))

We could collect 97 individuals of *M*. *rapidus* only from eight sites among 79 sites examined in this study. The presence sites were all in the Nam River system: 70 individuals from five sites in the main stream, 1 individual from one Im Stream site, 1 individual from one Yang Stream site, and 25 individuals from one Deokcheon River site. The presence sites were the main stream and tributary streams of the Nam and Deokcheon rivers, which flow into Jinyang Lake in the upper and middle regions of the Nam River. The main stream of the Nam River runs about 35 km, from Danseong-myeon to Saengcho-myeon of Sancheong-gun, wherein a relatively large group was observed at a site with rapids and a substrate of gravel, pebbles, and cobbles, and gently sloping pools with sand. One individual each was observed in the Im and Yang tributaries. At the Deokcheon River, 25 individuals were observed in the downstream rapids that flow into Jinyang Lake.

### Habitat features of presence sites

At presence sites, the RW, WW, and WD were 200–450 m, 100–200 m, and 0.1–1.2 m, respectively, in the lower to mid-lower reaches of rivers with pools and rapids areas with widespread gravel (Table 1). The AL, NFS, and NW were 42–128 m, 11–25, and 1 (St. 74), respectively. There were few weirs at these sites (Table 1). Apart from one site (St. 67) at Cheonghyeon Bridge in Mundae-ri, Sinan-myeon, Sancheong-gun, all sites were composed of sediment particles larger than sand (>60% gravel, pebbles, and cobbles). The water quality of the presence sites was relatively high. CON and DO were high, at 47.6–194.5 μS/cm and 9.8–10.7 mg/L. The pH was 6.9–8.9 (Table 1).

**Table 1.** Means and standard deviations (ranges) of variables measured in presence and absence habitats and results of the one-way ANOVA between the two different habitat types. Data from the eight sites (A) were used by average values in each site. Sample sizes of the three main sites (B) were 30 per variable per site.

Variables\Occurrence	Presence (*n* = no. measured places)	Absence (*n* = no. measured places)	*F*
**A. Eight sites**	(*n* = 8)	(*n* = 8)	
RW (m)	292.5 ± 87.3 (200–450)	291.6 ± 91.8 (130–400)	0
WW (m)	136.3 ± 35.0 (100–200)	121.9 ± 65.1 (50–225)	0.302
AL (m)	70.0 ± 36.8 (42–128)	42.9 ± 27.5 (3–90)	2.795
DO (mg/L)	10.4 ± 0.3 (9.8–10.7)	10.6 ± 0.5 (9.8–11.2)	0.702
CON (μs/cm)	133.9 ± 48.5 (47.6–194.5)	209.9 ± 50.7 (148.2–289.5)	9.425**
pH	8.2 ± 0.6 (6.9–8.9)	7.8 ± 0.3 (7.3–8.1)	1.637
WT (°C)	18.9 ± 3.2 (14.3–22.3)	16.2 ± 1.0 (14.8–17.4)	5.145*
SS (mg/L)	7.6 ± 2.2 (2.6–9.2)	9.8 ± 4.7 (6.5–20.3)	1.412
TN (mg/L)	1.6 ± 0.3 (1.2–1.9)	2.6 ± 0.8 (1.6–4.0)	10.20**
TP (mg/L)	0.04 ± 0.01 (0.04–0.05)	0.06 ± 0.04 (0.03–0.15)	1.523
NFS	19.1 ± 5.4 (11–25)	7.4 ± 3.9 (3–15)	25.37***
NW	0.3 ± 0.5 (0–1)	1.8 ± 1.4 (0–4)	8.40*
**B. Three main sites**	(*n* = 30)	(*n* = 30)	
WDP (cm)	61.4 ± 22.9 (25–115)	62.8 ± 35.6 (15–150)	0.864
LVP (m/s)	0.1 ± 0.1 (0.01–0.2)	0.2 ± 0.03 (0.01–0.5)	0.006**
MSP (cm)	9.6 ± 5.8 (0.1–18.0)	6.2 ± 6.9 (0.2–26.0)	0.045*
WDR (cm)	21.2 ± 5.6 (10–38)	23.5 ± 11.3 (8–50)	0.328
LVR (m/s)	0.4 ± 0.1 (0.2–0.6)	0.3 ± 0.1 (0.1–0.6)	0.062
MSR (cm)	7.1 ± 1.6 (3.2–11.8)	6.5 ± 4.7 (0.4–15.4)	0.529

Notes: Values are presented as mean ± SD. *P < .05, **P < .01, ***P < .001. Acronyms are RW (River width), WW (Water width), AL (Altitude), DO (Dissolved oxygen), CON (Conductivity), WT (Water temperature), SS (Suspended solids), TN (Total nitrogen), TP (Total phosphorus), NFS (Number of fish species), NW (Number of weir), WDP (Water depth at pool), LVP (Low velocity at pool), MSP (Mean substrate at pool), WDR (Water depth at ripple), LVR (Low velocity at ripple), MSR (Mean substrate at ripple).

### Comparison of habitat features between presence and absence sites

Of the 75 sites, the habitats of *M*. *rapidus* were confirmed at eight sites. However, these sites were all in the Nam River system, reflecting the limited distribution of *M*. *rapidus*. The results of the statistical significance test for the environmental factors of presence and absence habitat sites are shown in Table 1. Among the environmental factors, CON, WT, TN, NFS, and NW were significantly different between presence and absence sites (*P* < .05). The LV and MS at pools were significantly different in test for the rapids and pool areas (10 for each presence and absence site) (*P* < .05).

Four PCs with eigenvalues >1.0 were extracted from the PCA and applied to 10 environmental variables (Table 2). The PC3 scores significantly differed between the two sites (*F*_1,14_ = 7.094, *p* = .019; Figure 3(A)). PC3 explained 19.1% of total variance, with a high negative loading for NW and two positive loadings for pH and NFS. The second PCA applied to six variables and produced three PCs (Table 2). Only PC2 showed a significant difference between the two sites of fish occurrence (*F*_1,58_ = 15.150, *p* < .001; Figure 3(B)). PC2 explained 23.94% of total variance, with a high positive loading for MSP (Mean substrate at pool) and a negative loading for LVP (Low velocity at pool).

**Table 2.** Results of two principal component analyses, one based on eight sites (PCs 1–4), the other on three main sites (PCs 1–3) of presence and absence of *M. rapidus.* Correlations between original variables and the extracted PCs were shown only when >0.5.

	Loadings			
Variables	PC1	PC2	PC3	PC4
**A. Eight sites**				
RW		0.503		0.64
WW				0.872
AL		−0.665		
DO		0.799		
CON	0.726	0.507		
pH			0.651	0.622
SS	0.824			
TP	0.794			
NFS			0.604	
NW			−0.89	
Variance explained	22.67	19.346	19.115	17.302
Eigenvalue	2.267	1.935	1.912	1.73
**B. Three main sites**				
WDP	0.795			
LVP		−0.77		
MSP		0.816		
WDR	0.856			
LVR			0.922	
MSR	0.684			
Variance explained	32.07	23.94	18.03	
Eigenvalue	1.924	1.436	1.082	

Note: Acronyms are RW (River width), WW (Water width), AL (Altitude), DO (Dissolved oxygen), CON (Conductivity), SS (Suspended solids), TP (Total phosphorus), NFS (Number of fish species), NW (Number of weir), WDP (Water depth at pool), LVP (Low velocity at pool), MSP (Mean substrate at pool), WDR (Water depth at ripple), LVR (Low velocity at ripple), MSR (Mean substrate at ripple).

## Discussion

**Figure 2.**
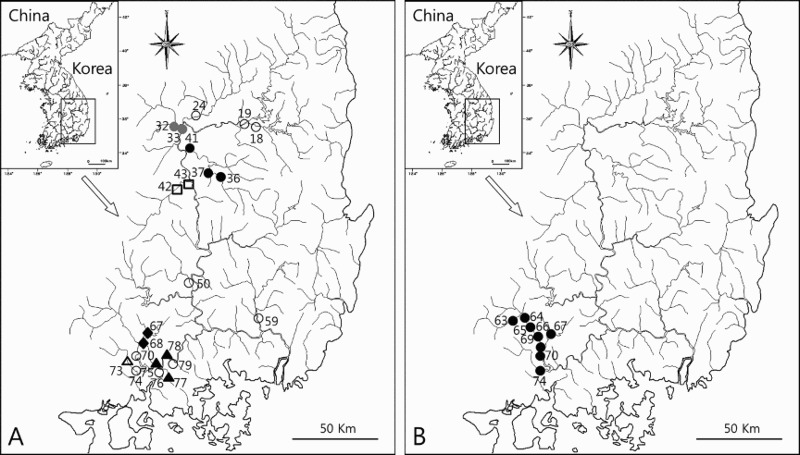
Presence sites of the *M*. *rapidus* in south-eastern Korea from 1996 to 2014. A: Open circle; Chae et al. (1996), Gray circle; Chae et al. (1998a), Black circle; Chae et al. (1998b), Open triangle; Lee & Kim (2002), Black triangle; Kum & Yang (2002), Open square; Kang et al. (2004); Black diamond; Ministry of environment of Korea (2008), B: Black circle; Present study. Data of sites is shown in Supplementary Table S1.

When comparing the distribution status of *M*. *rapidus* from 1996 to 2008 with the current presence sites (Chae et al. 1996, 1998a, 1998b; MEK 1997–2008; Kum & Yang 2002; Lee & Kim 2002; Kang et al. 2004), we found that *M*. *rapidus* inhabited the main stream and tributary stream of the Nakdong River and presumed that its population was greatly decreased or became extinct, based on the survey of the Ministry of Environment in 2008 demonstrating that its habitats were only in the Sancheong-gun district of Gyeongsangnam-do (Figure 2). At the Yeong River, which was among the sites where >30 individuals of *M*. *rapidus* were collected, individuals were reported to inhabit downstream rapids with gravel near the confluence of the Nakdong River, but this was not confirmed by the present study. Since 2010, the riparian ecosystem has seen considerable changes, including the disappearance of rapids and the transformation of lotic areas into lentic ones as a result of construction of the Four Major Rivers Project targeting the Han, Nakdong, Geum, and Yeongsan Rivers. *M*. *rapidus* is unlikely to inhabit the Yeong River as long as the lentic conditions persist (Chae et al. 2014). Kang et al. (2004) reported that the most habitats were found in St. 42, where the substrate was mostly sand. Weirs were built across the river at the confluence with the Nakdong River, and the pier construction was in progress under the weirs. The level of artificial river disturbance was severe. Therefore, it is presumed that *M*. *rapidus* is unlikely to inhabit this area due to the reduced flow velocity caused by the weirs and the continuous accumulation of sand from upstream. Chae et al. (1996) collected >30 individuals from the Nam River, downstream of Jinyang Lake. However, this species, which prefers relatively high water quality, may no longer be able to inhabit this area due to the deterioration of water quality caused by plastic film houses and agricultural facilities, coupled with riverbed disturbances from repeated flood damage rehabilitation projects. Apart from the above three areas, continuous riverbed disturbance also occurs in Wi, Banbyeon, Naeseong, and Hoe streams and Miryang River due to water pollution and river renovation projects, likely making these areas uninhabitable for *M*. *rapidus*. However, both past and current distribution surveys have confirmed the presence of *M*. *rapidus* in the Deokcheon and Nam river systems flowing into Jinyang Lake. In these areas, high-quality stream water is available all year round. In addition, St. 64, St. 70, and St. 74, which form a relatively large group, are in a protected area that prevents artificial river development and renovation. This may enable *M*. *rapidus* to inhabit these areas.

**Figure 3.**
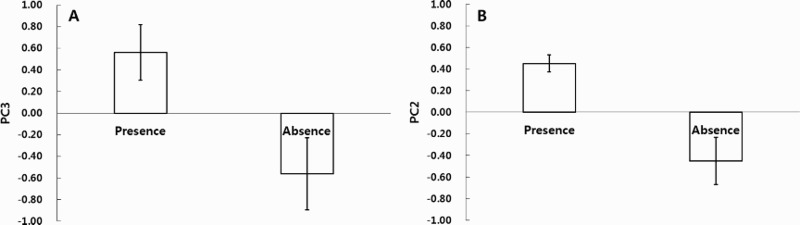
Mean (± SE) values of principal component 3 (PC3) from eight sites (A) and principal component 2 (PC2) from three main sites (B), where individual scores are coded and compared according to whether their sites show the presence or absence of *M*. *rapidus*; it is evident that the two sites differ markedly in their scores on PC3 of (A) as well as on PC2 of (B).

The sites where *M*. *rapidus* was common had a high WT and NFS, and low CON, TN, and NW. Among these, NFS was the most different between presence and absence sites (*P* < .001). Various habitat characteristics, including available shelter, spawning grounds, and prey, are required to accommodate many different species of fishes. The sites where *M*. *rapidus* was absent have had many flood damage rehabilitation and river renovation projects since 2000. Since 2010, the Four Major Rivers Project has led to the loss of riverbed materials and rising water levels at tributary streams, up to 6 m at the main stream of the Nakdong River. Such projects have transformed many habitats into lentic ecosystems, decreasing the number of rapids-preferred and benthic fish species, increasing the number nekton fishes (Chae et al. 2014), and reducing the NFS overall. However, areas upstream of the sites where *M*. *rapidus* was present were within protected areas, and had a lower NW (or none at all), which helped to maintain the natural state of the river and retain critical fish habitat, resulting in a high NFS. In addition, CON and TN at the presence sites were also significantly different from those at the absence sites (*P* < .01). CON generally increased proportionally with the number of ionic contaminants. Absence sites tended to be in or near large cities or other areas with inflow of pollutants from point and non-point sources (e.g. farmlands and stables), which are likely to increase CON. On the other hand, clean water in the Nam River system was provided all year round at the presence sites, which likely results in low CON. An et al. (2012) reported that elevated CON, caused by point and non-point pollutant sources and the inflow of domestic sewage from urban housing complexes, did not have a direct impact on fish populations, but in the long term may have adverse effects on tolerance, the trophic guilds of fishes, and fish health. The inflow of nutrients such as TN can have a significant impact on the number of fish species and population size, shifting towards a community structure dominated by pollution-tolerant and omnivorous species (Kim et al. 2012). Water quality is clearly an important restrictive factor for fishes, demonstrated by the presence of *M*. *rapidus* in the Nam River and its absence from the Deokcheon River. This is demonstrated by the results of the PCA for the presence and absence sites with 10 variables, which indicates that higher NFS and pH and lower NW were associated with the presence of *M*. *rapidus* (Table 2 and Figure 3(A)). According to the results of PCA for the main presence and absence sites with six physical variables, lower LVP and higher MSP were associated with the presence of *M*. *rapidus* (Table 2 and Figure 3(B)). It is presumed that the low water velocity and the presence of pebbles and cobbles in the substrate provide shelter and habitat for juvenile fish, which may increase survival and recruitment. However, an analysis of the relationship between these microhabitats and life history is needed for a more definited assessment.

The IUCN Red List criteria for selecting and protecting endangered species include the number and size of populations, area of occupancy, and the tendency of these factors to change (IUCN 2001). In the Red Data Book of Endangered Species of Wild Fauna and Flora in Korea, *M*. *rapidus* is evaluated based on occupancy information only, due to the lack of data on population size (NIBR 2011). We identified eight new sites of occurrence. However, only one individual was observed at three of those sites, namely, the Im Stream (St. 63), Nam River (St. 65), and Yang Stream (St. 67). We considered these individuals to have been migrants rather than residents, thus placing the distribution of *M*. *rapidus* into the severely fragmented category, with less than five known locations. An analysis using the program Geospatial Conservation Assessment Tool (GeoCAT; richmond, London, UK) revealed that the extent of occurrence and area of occupancy were less than 5000 km^2^ and 500 km^2^, respectively. This indicates that the extent or quality of habitats and number of areas of *M*. *rapidus* have decreased compared to previous findings, and thus this species can be considered to qualify for the endangered B2ab (i, ii, iii, iv, v) classification of the Red List, meaning that it is critically endangered in the wild. A previous study (Chae et al. 1996, 1998a, 1998b; MEK 1997–2008; Kum & Yang 2002; Lee & Kim 2002; Kang et al. 2004) reported a total of 22 sites of occurrence, but we found only 8, a decrease by 63.6%. Records from the last 10 years indicate that this species appeared at two sites in the Yang Stream, which we did not find. However, it is difficult to compare these distribution statuses because a detailed distribution survey for *M*. *rapidus* has not been conducted in the last decade. Moreover, because the eight sites observed in the present study were all related to some part of the Nam and Deokcheon River systems, we can confirm the rapid decrease in *M*. *rapidus* populations that had once been widespread throughout the Nakdong River system.

The presence distribution survey indicated that the Deokcheon River group in the Nakdong River system had few downstream populations due to the rehabilitation projects and weir construction. However, it was confirmed through continuous monitoring that the population of *M*. *rapidus* at St. 64 is relatively stable. Therefore, various methods of *in situ* conservation need to be established, including the elimination of extinction threats from the natural habitats at St. 64, St. 70, and St. 74 (which form relatively stable populations), and the designation of the coastal zone from St. 63 to St. 64 (which is estimated to have the largest population) as a legally protected area. In addition, based on the confirmed distribution and habitat features, conservation studies using continuous monitoring, fundamental ecological research, and genetic diversity analyses are required.
